# Impacts of Spatial Resolution and XCO_2_ Precision on Satellite Capability for CO_2_ Plumes Detection

**DOI:** 10.3390/s24061881

**Published:** 2024-03-15

**Authors:** Zhongbin Li, Meng Fan, Jinhua Tao, Benben Xu

**Affiliations:** 1 State Key Laboratory of Remote Sensing Science, Aerospace Information Research Institute, Chinese Academy of Sciences, Beijing 100101, China; lizhongbin@aircas.ac.cn (Z.L.); taojh@aircas.ac.cn (J.T.); xubb@aircas.ac.cn (B.X.); 2 School of Electronics and Information, Northwestern Polytechnical University, Xi’an 710072, China

**Keywords:** anthropogenic CO_2_ emissions, satellite design, Gaussian plume model, spatial resolution, XCO_2_ precision

## Abstract

Greenhouse gas satellites can provide consistently global CO_2_ data which are important inputs for the top-down inverse estimation of CO_2_ emissions and their dynamic changes. By tracking greenhouse gas emissions, policymakers and businesses can identify areas where reductions are needed most and implement effective strategies to reduce their impact on the environment. Monitoring greenhouse gases provides valuable data for scientists studying climate change. The requirements for CO_2_ emissions monitoring and verification support capacity drive the payload design of future CO_2_ satellites. In this study, we quantitatively evaluate the performance of satellite in detecting CO_2_ plumes from power plants based on an improved Gaussian plume model, with focus on impacts of the satellite spatial resolution and the satellite-derived XCO_2_ precision under different meteorological conditions. The simulations of CO_2_ plumes indicate that the enhanced spatial resolution and XCO_2_ precision can significantly improve the detection capability of satellite, especially for small-sized power plants with emissions below 6 Mt CO_2_/yr. The satellite-detected maximum of XCO_2_ enhancement strongly varies with the wind condition. For a satellite with a XCO_2_ precision of 0.7 ppm and a spatial resolution of 2 km, it can recognize a power plant with emissions of 2.69 Mt CO_2_/yr at a wind speed of 2 m/s, while its emission needs be larger than 5.1 Mt CO_2_/yr if the power plant is expected to be detected at a wind speed of 4 m/s. Considering the uncertainties in the simulated wind field, the satellite-derived XCO_2_ measurements and the hypothesized CO_2_ emissions, their cumulative contribution to the overall accuracy of the satellite’s ability to identify realistic enhancement in XCO_2_ are investigated in the future. The uncertainties of ΔXCO_2_ caused by the uncertainty in wind speed is more significant than those introduced from the uncertainty in wind direction. In the case of a power plant emitting 5.1 Mt CO_2_/yr, with the wind speed increasing from 0.5 m/s to 4 m/s, the simulated ΔXCO_2_ uncertainty associated with the wind field ranges from 3.75 ± 2.01 ppm to 0.46 ± 0.24 ppm and from 1.82 ± 0.95 ppm to 0.22 ± 0.11 ppm for 1 × 1 km^2^ and 2 × 2 km^2^ pixel size, respectively. Generally, even for a wind direction with a higher overall uncertainty, satellite still has a more effective capability for detecting CO_2_ emission on this wind direction, because there is more rapid growth for simulated maximal XCO_2_ enhancements than that for overall uncertainties. A designed spatial resolution of satellite better than 1 km and a XCO_2_ precision higher than 0.7 ppm are suggested, because the CO_2_ emission from small-sized power plants is much more likely be detected when the wind speed is below 3 m/s. Although spatial resolution and observed precision parameters are not sufficient to support the full design of future CO_2_ satellites, this study still can provide valuable insights for enhancing satellite monitoring of anthropogenic CO_2_ emissions.

## 1. Introduction

The Intergovernmental Panel on Climate Change (IPCC) notes that global greenhouse gas emissions have risen to an unprecedented level, with a linear increase in the average temperature of the global land and ocean surface [1]. The impacts of climate change have led to irreversible effects on agriculture, ecological systems, and terrestrial and marine environments. The Paris Agreement is committed to substantially reducing global greenhouse gases (GHGs) emissions with the objective of maintaining the global temperature increase within this century below 2 °C above pre-industrial levels, while pursuing effects to limit the temperature increase to 1.5 °C [2]. As the main component of atmospheric GHGs, carbon dioxide (CO_2_) accounts for approximately 65% of the radiation forcing from long-lived GHGs [3]. In 2021, Human activities were responsible for the emission of about 37.12 billion metric tons of CO_2_, predominantly from the combustion of fossil fuels in industrial processes [4]. Atmospheric CO_2_ measured at NOAA’s Mauna Loa Atmospheric Baseline Observatory peaked for 2021 in May at a monthly average of 419 parts per million (ppm), the highest level since accurate measurements began 63 years ago, scientists from NOAA and Scripps Institution of Oceanography at the University of California San Diego announced today. Emission hot spots, such as megacities, large steel producing factories, volcanoes, and power plants, are major contributors to global energy-related CO_2_ emissions [5,6]. Compared to other point sources, power plants are more representative, power plants contribute a significant portion of global greenhouse gas emissions. They have continuous emission durations, stable emission rates, and emissions that are more concentrated. Additionally, monitoring the emission rates of power plants is already well-established, with emission inventories providing emission rate information. Although all are considered point sources of emissions, power plants better fit the theoretical definition of a point source. Consequently, the quantification and routine update of global CO_2_ emissions of these hot spots are crucial for the international climate negotiations focused on carbon emission reduction.

The top-down and bottom-up approaches are two primary methodologies of CO_2_ emission estimation. The bottom-up approach provides detailed information on individual sources, whereas the top-down approach yields more general information on overall emissions. Bottom-up CO_2_ emission inventories are often subject to significant uncertainties due to reasons such as the limitations in data availability and adequacy, as well as the techniques employed in data processing. With the development of GHG satellites in recent years, space-based observations are increasingly used for the top-down inverse estimation of CO_2_ emission. This approach estimates regional carbon sources and sinks and their dynamic changes by combining atmospheric observations of greenhouse gas concentrations and meteorological data with atmospheric chemical transport models through complex data assimilation methods. Satellite observations have an advantage over ground-based measurements by providing comprehensive and consistent atmospheric CO_2_ data for both terrestrial and marine surfaces on a global scale. Presently, numerous countries are committed to developing global or regional Monitoring and Verification Support (MVS) systems for anthropogenic CO_2_ emissions, in alignment with the Paris Agreement of the United Nations Framework Convention on Climate Change (UNFCCC). These MVS systems are designed to enhance the accuracy of national greenhouse gas emissions inventories, particularly through top-down verification of emissions using globally high spatial and temporal resolution observations from space-based sensors [5,6,7]. For instance, a European Commission (EC) expert panel raised high-level requirements that have been translated into technical requirements, proposing technical specifications for satellite sensors with clearly defined spatial and temporal resolutions and emission source monitoring capabilities [3].

Several countries, including Japan, the United States, and China, have launched GHG satellites [8,9,10,11,12]. The Greenhouse Gases Observing Satellite (GOSAT) as Japan’s pioneering GHG satellite was launched in January 2009. It was designed to monitor the global distributions of CO_2_ and CH_4_ from space and remains operational. As a successor mission of GOSAT, GOSAT-2 equipped with the improved TANSO-FTS-2 featuring enhanced signal-to-noise ratio (SNR), was launched in October 2018. Both GOSAT and GOSAT-2 employ non-consecutive sampling, cross-track mechanical pointing, which restrict their observational spatial coverage (swath 790 km, 10.5 km at sub-satellite point) [13]. Launched in July 2014, Orbiting Carbon Observatory-2 (OCO-2) is an Earth observing satellite mission owned and operated by National Aeronautics and Space Administration (NASA). The OCO-2 instrument utilizes a pushroom scanning technique with a swath width of 10 km and spatial resolution of 1.29 km cross-track and 2.25 km along-track [14]. In December 2016, China successfully launched its first GHG observation satellite, TanSat. OCO-2 and Transat use continuous pixel observations facilitated by high-resolution raster spectroscopy, enabling monitoring CO_2_ emissions from point sources [15]. However, due to their narrow swath widths (10~25 km) and revisit periods, there is a notable observational gap area between their orbital paths, limiting their ability to capture temporal variability of emissions from sources like power plants [16]. OCO-3, installed on the International Space Station (ISS) in May 2019, introduced Snapshot Area Map mode, targeting specific areas approximately 80 × 80 km^2^, such as fossil fuel emission sources, volcanoes, and field validation sites. Through multiple observations of the same power plant by OCO-3, this mode assists in quantifying complex emission sources [5,17]. Above operational satellites (GOSAT, GOSAT-2, OCO-2, OCO-3 and TanSat) represent the first generation of CO_2_ satellite missions, primarily focused on obtaining global CO_2_ distribution. By using long time-series XCO_2_ monitored by these satellites, it can offer insights into continental-scale CO_2_ budgets and initial understanding of the carbon flux in land surface systems [17,18,19,20]. Atmospheric CO_2_ concentration has been examined in several studies from different perspectives, such as spatial distribution and relation with different variables and so on [13,21,22,23]. While the detection accuracy of these satellites has improved to a certain level, their overall capabilities remain limited [6,24], particularly in terms of coverage range and spatial-temporal resolution. Therefore, in view of the technical shortcomings of the first generation of carbon satellites, the detection capability of the next generation of carbon satellites needs to be improved, and not only the spatial and temporal resolution, but also the coverage range.

To meet the requirements for CO_2_ emissions monitoring and verification support capacity, the next-generation CO_2_ satellites is being conceptualized and developed by the European Union (EU), Japan, the United States, China, and so on. The Copernicus Anthropogenic Carbon Dioxide Monitoring (CO2M), as a part of the EU’s Copernicus Sentinel Expansion mission, is specifically designed to quantify CO_2_ emissions attributable to human activities. Planned for launch in 2025, CO2M aims to enhance our understanding of anthropogenic CO_2_ release. Meanwhile, China is progressing with its next-generation CO_2_ satellite mission, which is currently undergoing a rigorous evaluation of satellite platform and payloads designing. The next-generation CO_2_ satellites are tasked with reducing current uncertainties in estimation of CO_2_ emissions from fossil fuel combustion at both national and regional levels. Given the rapid dilution of CO_2_ after emission, further satellites are expected to possess enhanced capabilities for frequent and comprehensive capture of entire CO_2_ plumes with high single-sounding precision [25]. These above requirements translate into needs for the XCO_2_ retrieval precision, spatial sampling, spatial resolution, and global coverage. These prerequisites further drive the design of satellite instruments, particularly concerning SNR, as well as spatial co-registration and spectral stability. Such developments are essential for achieving the desired accuracy and efficiency in CO_2_ emission monitoring and verification at a global scale.

For the next-generation CO_2_ satellite missions design, two pivotal observational characteristics for detecting plumes from coal-fired power plants are spatial resolution and the precision of satellite-derived XCO_2_ measurements. The detectable signal of enhanced XCO_2_ also depends on the wind field due to the rapid dispersion of CO_2_ post-emission. In this study, the Gaussian plume model method was used to simulate and analyze the CO_2_ diffusion under a variety of atmospheric conditions and for different emission sources, considering the impact of varying pixel sizes on satellite monitoring effectiveness. Section 3 presents a quantitative analysis of the relationship between spatial resolution and satellite monitoring capability for terrestrial CO_2_ emission sources under different atmospheric conditions and emission intensities. In Section 4, we explore and discuss the effects of the simulated wind field, satellite-derived XCO_2_, and hypothesized CO_2_ emission on the overall uncertainty of realistic XCO_2_ enhancements which can be identified by satellite. Although the results of this study alone are not definitive in determining the design architecture of future CO_2_ monitoring satellites, they provide valuable insights that may guide the development of space-based systems for monitoring anthropogenic CO_2_ emissions.

## 2. Methods

### 2.1. Simulation Region

In this study, to assess the efficacy of satellite-based monitoring of CO_2_ emissions from power plants, simulations were conducted to analyze XCO_2_ enhancements under different scenarios within a defined area of 60 × 60 km^2^. This designated region was subdivided into 50 m × 50 m grids for the generation of realistic CO_2_ plume simulations. The XCO_2_ enhancement data derived from these simulations were further averaged across these grid-generated CO_2_ plume images. To mimic satellite observations, these data were further utilized to create downscaled square tiles, simulating satellite observation images at spatial resolutions of 0.5 × 0.5 km^2^, 1 × 1 km^2^, 2 × 2 km^2^, and 4 × 4 km^2^. Uncertainties in the XCO_2_ enhancement identified by satellite were statistically analyzed. This analysis considered various factors, including meteorological conditions, CO_2_ emissions and satellite noises, to evaluate the precision and reliability of satellite observations in monitoring atmospheric CO_2_ levels.

### 2.2. Gaussian Plume Model

Currently, the Gaussian plume model is mainly used for estimating the emission of a single power plant using satellite observations of XCO_2_ [15,26]. For the simulation of XCO_2_ enhancement above the background concentration, an advanced version of the Gaussian plume model was adopted in this study [27], specifically an improved vertically integrated Gaussian plume model. This refined model is predicated on a series of slightly modified equations used by Bovensmann et al. [28]. These modifications enhance the model’s accuracy and applicability in simulating the dispersion of XCO_2_ in the atmosphere, providing a more precise representation of CO_2_ plume behavior.

(1) $$V(x,y)=\frac{F}{\sqrt{2\pi }\sigma_{y}(x)\mu }e^{-\frac{1}{2}{\left(\frac{y}{\sigma_{Y}(x)}\right)}^{2}}$$

(2) $$\sigma_{y}(x)=a{\left(\frac{x}{x_{0}}\right)}^{\gamma }$$

where *V* (*x*, *y*) is the CO_2_ vertical column density (in g/m^2^) at coordinates (*x*, *y*). Here, *x* (in m) denotes the downwind distance from the point source, and *y* (in m) is the distance perpendicular to the wind. The orientation of *x* is parallel to the wind direction, and the orientation of *y* is perpendicular to the wind direction. The wind direction (θ) is measured counterclockwise from the eastward direction to the along-track direction of satellite. *V* (*x*, *y*) can be calculated from CO_2_ emission rate (*F*, in g/s), wind speed (*μ*, in m/s), and the standard deviation in the *y* direction (*σ_y_*(*x*), in m) which determines the rate of diffusion perpendicular to the wind. *a* (in m) is the atmospheric stability parameter, determined based on the Pasquill–Gifford stability classification [29,30]. Here, *a* is depending on Pasquill stability classes, which can be determined from the 10 m wind speed and solar radiation. *a* is empirically set at different values based on wind speed: *a* = 213 m for *μ <* 2 m/s, either *a* = 213 or 156 m for 2 m/s ≤ *μ <* 3 m/s, *a* = 156 m for 3 m/s ≤ *μ <* 5 m/s, and *a* = 104 m for *μ* > 5 m/s [29,30]. Additionally, the previous Gaussian dispersion model did not consider the influence of crosswind diffusion when calculating *σ_y_*(*x*). Therefore, later scholars added crosswind diffusion to the original formula, namely: *x*_0_ = 1000 m, where *x*_0_ is defined as the characteristic length representing crosswind diffusion. The parameters set in the formulas are only for the simulation of plume dispersion in the next-generation carbon satellite. These parameters are universal, but meteorological factors in actual pollution processes are constantly changing. Therefore, the results simulated using the parameters set in the document may not fully represent the complete pollution dispersion process under real conditions.

Then, the enhancement of XCO_2_, quantified in parts per million (in ppm), is derived from *V* (*x*, *y*) based on the following equation [26,31]:

(3) $$XCO_{2}=V(x,y)\cdot \frac{M_{air}}{M_{CO_{2}}}\cdot \frac{g}{P_{surf}-w\cdot g}\cdot 1000$$

where *M_air_*, *M_CO_*_2_, and *g* are three constants. *M_air_* and *M_CO_*_2_ represent the molecular weights (in kg/mol) of air and CO_2_, respectively. And *g* denotes the gravitational acceleration (in m/s^2^). *P_surf_* refers to the surface pressure (in Pa), and *w* signifies the total column water vapor (in kg/m^2^).

The background wind field in this study is assumed to be stable and uniform, ensuring that the XCO_2_ distribution adheres to a normal distribution pattern throughout the continuous point source diffusion process. It is important to note that the CO_2_ plume is considered only in terms of horizontal dispersion, with turbulent diffusion along the wind direction (*θ*) being disregarded, and the vertical diffusion of the plume being ignored. Therefore, the influence of terrain effects on plume dispersion was not considered.

### 2.3. Uncertainties Estimation

The capacity of satellites to detect CO_2_ plumes emitted from point sources, such as power plants, is intrinsically related to several key factors: the simulated wind field, the satellite-derived XCO_2_ measurements, and the hypothesized CO_2_ emissions. Point source emissions dispersion is greatly influenced by wind. Wind speed affects the speed of pollutant spread, while wind direction affects the direction of pollutant spread. The plume enhancement results captured by satellites include errors inherent to the satellites themselves, which can impact the plume enhancement results to a certain extent. The assumed CO_2_ emission results come from ground monitoring devices, which have their own monitoring errors, leading to errors in CO_2_ emission estimates. Consequently, the uncertainties or errors inherent in these three factors cumulatively contribute to the overall accuracy of the satellite’s ability to identify realistic enhancement in XCO_2_. This overall uncertainty plays a pivotal role in determining the reliability and precision of satellite-based observations in recognizing atmospheric CO_2_ variations.

Through tuning parameters such as wind speed, wind direction, satellite-derived XCO_2_ error, and the uncertainty of CO_2_ emission from the power plant, the overall uncertainty in satellite-detected XCO_2_ enhancement (denoted as *ε*) can be quantitatively determined by

(4) $$\varepsilon =\sqrt{\varepsilon_{w}^{2}+\varepsilon_{s}^{2}+\varepsilon_{e}^{2}}$$

where *ε_w_* represents the simulated uncertainty associated with the wind field, which is determined as the standard deviation derived from incorporating uncertainties into both wind speed and wind direction for plume simulation. *ε_s_* is attributed to the error in satellite-based XCO_2_ led by the instrument noise and the model error of XCO_2_ retrieval. Furthermore, *ε_e_* is the discrepancy of CO_2_ emissions, contrasting the statistical data from “bottom-up” CO_2_ emission inventory with the actual emission values. *ε* is the total error then determined from the wind, instrument noise, and CO_2_ emissions.

Here, *ε_w_* can be calculated by

(5) $$\varepsilon_{w}=\sqrt{\varepsilon_{w}^{2}\left(\mu \right)+\varepsilon_{w}^{2}\left(\theta \right)}$$

where *ε_w_*(*μ*) and *ε_w_*(*θ*) are the uncertainties in XCO_2_ introduced by wind speed and wind direction, respectively.

## 3. CO_2_ Plume Simulation and Analysis

### 3.1. CO_2_ Emission from Plant Power

**Figure 1:**
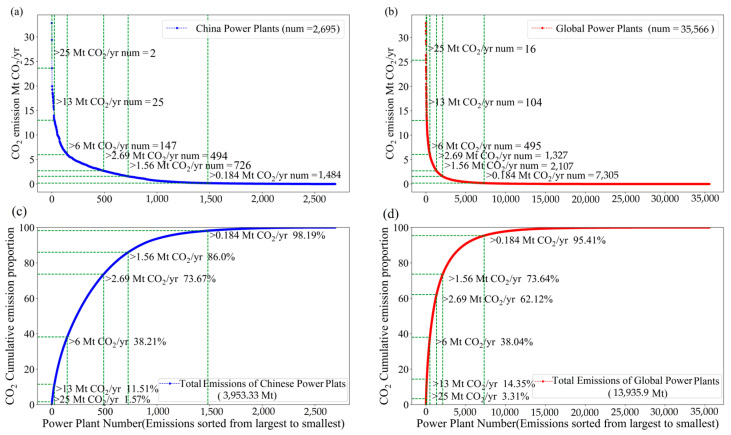
Statistics of power plant number (**a**,**b**) and cumulative emission (**c**,**d**) in 2019 from GPED v1.1. Left column is for power plants in China, and right column is for global data.

As the most important point source, CO_2_ emissions from power plants constitute a significant portion of global anthropogenic CO_2_ sources. In order to evaluate the satellite detection capability of power plants around the world and China, the global power emissions database (GPED) v1.1 was used for counting the CO_2_ emission ratio of the satellite-detected amount to the total amount from power plants. GPED is a publicly available bottom-up emission inventory including global plant-level CO_2_ emission data of power plants across all countries [32]. Analysis of 2019 emission statistics from GPED v1.1, as illustrated in Figure 1, reveals that power plants with CO_2_ emissions of more than 0.184, 6, 13 and 25 Mt CO_2_/yr comprise only 20.53%, 13.91%, 0.29% and 0.04% of the global total count, yet their cumulative emissions account for 95.41%, 38.04%, 14.35% and 3.31% of the total amount of CO_2_ emission from global power plants, respectively. In the context of China, power plant emissions over 0.184, 6, 13 and 25 Mt CO_2_/yr correspond to 55.06%, 5.45%, 0.92% and 0.07% of the total number, contributing 98.19%, 38.21%, 11.51% and 1.57% of the country’s total CO_2_ emission, respectively. Here, the reference CO_2_ emission level of 0.184 Mt CO_2_/yr is one of the high-level policy needs to develop the MVS capacity for anthropogenic CO_2_ emissions in the EU, as proposed by Pinty et al. [33]. Furthermore, according to previous studies, emission levels of 6, 13 and 25 Mt CO_2_/yr can be employed to categorize low-, medium- and high-sized power plants, respectively [15].The emission rates of power plants in reality are subject to variations due to hardware and external factors, and are not constant. This article simply uses constant emission rates for plume simulation under ideal conditions.

### 3.2. Detection Capability of Satellite with Different Spatial Resolutions and XCO_2_ Precisions

To assess the performance of satellite in the detection of CO_2_ plumes from power plants with different CO_2_ emission intensities, 6, 13, and 25 Mt CO_2_/yr were converted to CO_2_ emission rate *F* to represent the low, medium, and high emission level of point sources, respectively. *μ =* 4 m/s, *a =* 156.0 m, *θ* = 45° were set for the simulation of CO_2_ plume over a domain with a pixel size of 0.5 × 0.5 km^2^. Based on existing research findings and the latest XCO_2_ measurement results, the background concentration of XCO_2_ in this experiment is set to 420 ppm. This value represents an idealized state and is considered universally applicable, without accounting for regional variations. It is solely used for simulating payloads for the next-generation carbon satellite.

**Figure 2:**
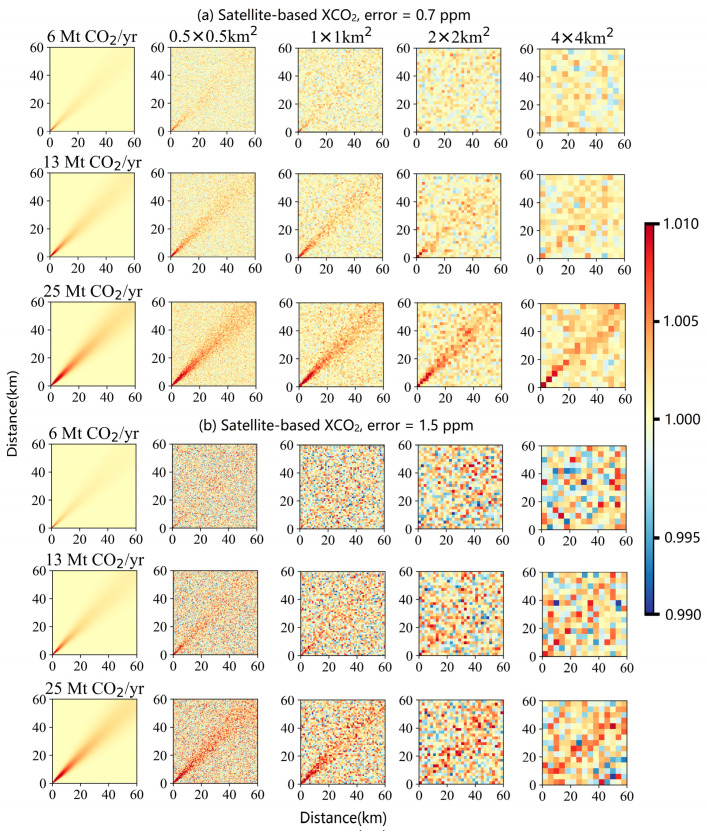
Plume simulations of XCO_2_ observations relative to a constant 420 ppm background for satellite-based XCO_2_ error of 0.7 ppm (**a**) and 1.5 ppm (**b**). Column 1 shows the simulated Gaussian plume at 50 m resolution for each power plant emission rate against a clean background. Columns 2~5 are simulated observations with a spatial resolution of 0.5, 1, 2 and 4 km, respectively. Top to bottom rows in each panel are simulated observations for CO_2_ emission of 6, 13, and 25 Mt CO_2_/yr, respectively.

Additionally, the space-based XCO_2_ error caused by instrument noise and retrieval algorithm is also a primary limitation in the detection of CO_2_ plumes. The XCO_2_ monitoring precision for the first-generation CO_2_ satellites (e.g., GOSAT, GOSAT-2, OCO-2 and Tansat) is ~1.5 ppm [8]. In contrast, the next-generation CO_2_ satellites (e.g., CO2M and Tansat2) aim for a more stringent XCO_2_ precision of ~0.7 ppm. Noise was superimposed over the simulated enhancements to represent random errors that would occur in true observations. The noise or random error was generated following a normal distribution. Here, to simulate realistic satellite observations with different spatial resolutions, gaussian errors of 0.7 and 1.5 ppm (i.e., *ε_s_*) were added to generate simulated images of satellite observation with different spatial resolutions. The outcomes of these simulations, showcasing the impact of these errors on observational accuracy at different spatial resolutions, are depicted as shown in Figure 2.

According to the simulated XCO_2_ enhancement results, it is indicated that for the power plant with low-level emission of 6 Mt CO_2_/yr, identifying the emitted CO_2_ plume becomes challenging when the spatial resolution is coarser than 1 km. This is primarily because the XCO_2_ enhancement from emission is either smaller than or comparable to the XCO_2_ error from satellite observation. A notable increase in the capability to detect CO_2_ plumes from space is shown when the *ε_s_* is reduced from 1.5 ppm to 0.7 ppm. For satellites with an *ε_s_* of 1.5 ppm, the substantial noise interference significantly weakens the CO_2_ plume signal, making it discernible only for emission sources with 25 Mt CO_2_/yr at pixel sizes of 0.5, 1 and 2 km. However, for *ε_s_* = 0.7 ppm, power plants emitting more than 13 Mt CO_2_/yr emission can be effectively detected by satellite through clearly observable CO_2_ plumes at spatial resolutions of 4 km or higher. Even though a distinct shape of CO_2_ plume may not be obtained for a simulation region with 1 × 1 km^2^ and lower emission rates, a remarkable XCO_2_ enhancement in several specific pixels proximal to the emission source can still be detected by satellite with an *ε_s_* = 0.7 ppm. And such isolated pixels characterized by abnormally high XCO_2_ values also have potential for estimating CO_2_ emissions from point sources through multiple repeat observations by satellite. Therefore, compared with the first-generation CO_2_ satellites, the indicator improvement in XCO_2_ precision will enhance the detection of the CO_2_ emission plumes and isolated high-level XCO_2_ pixels for the next-generation CO_2_ satellites, thereby benefiting the capturing of point source emissions. The set satellite monitoring precision and the simulated plume dispersion results at different spatial resolutions in this study are all aimed at meeting the observation requirements of the next-generation carbon satellite. These simulations serve as a forward simulation for determining satellite payload indicators.

**Figure 3:**
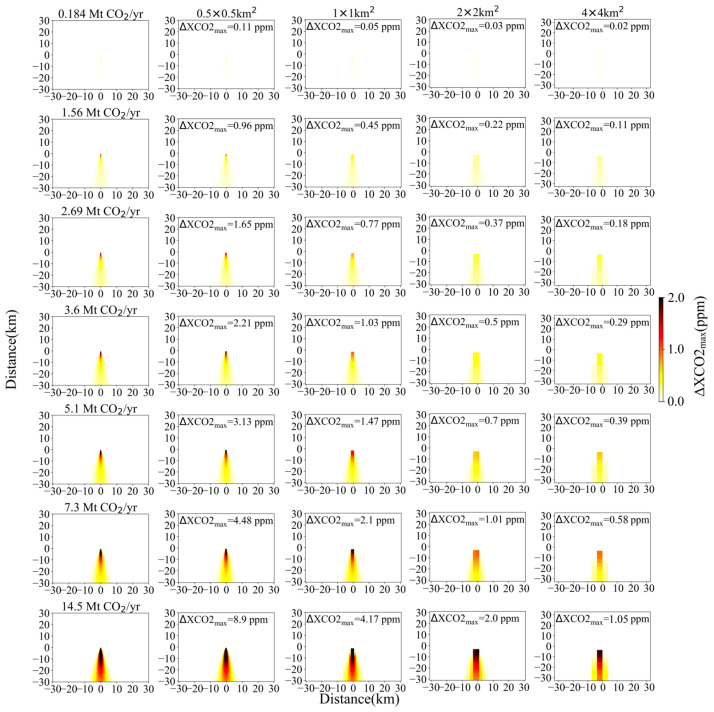
XCO_2_ enhancement distribution and ΔXCO2_max_ simulations with *μ* = 4 m/s and *θ* = 270°. Column 1 shows the simulated Gaussian plume at 50 m resolution for each power plant emission rate against a clean background. Columns 2~5 are simulated observations with a spatial resolution of 0.5, 1, 2 and 4 km, respectively. Top to bottom rows are simulated observations for CO_2_ emissions of 0.184, 1.56, 2.69, 3.6, 5.1, 7.3 and 14.5 Mt CO_2_/yr, respectively.

To investigate the detection capability of satellite more quantitatively for CO_2_ emissions from small- and medium-sized power plants (emissions < 15 Mt CO_2_/yr), we conducted a series of plume simulations with a 2 × 2 km^2^ pixel size. These CO_2_ plume simulations were generated by changing CO_2_ emissions in a range of 0~15 Mt CO_2_/yr with a step of 0.1 Mt CO_2_/yr. Here, taken 2 × 2 km^2^ as a standard value, the main reason is that 2 km is the designed spatial resolution of both CO2M and Tansat2. As shown in Figure 3, under conditions of *μ* = 4 m/s and *θ* = 270°, if the maximum values of XCO_2_ enhancement (donated as ΔXCO2_max_) in the simulation domain reach up to 0.5, 0.7, 1.0 and 2.0 ppm, the source emissions need to be larger than 3.6, 5.1, 7.3 and 14.5 Mt CO_2_/yr, respectively. It is indicated that the *ε_s_* of satellite should be less than 0.5, 0.7, 1.0 and 2.0 ppm, so that the XCO_2_ enhancements generated by power plants with an emission of 3.6, 5.1, 7.3 and 14.5 Mt CO_2_/yr can be detected, respectively. In addition, considering that power plants with emissions larger than 1.56 or 2.69 Mt CO_2_/yr contribute ~85% and ~75% to the total emissions of global power plants based on GPED (Figure 1), emission levels of 1.56 and 2.69 Mt CO_2_/yr were also selected as reference emission thresholds with the other four values mentioned above in Section 3.1.

Based on the simulations depicted in Figure 3, satellites with higher spatial resolution can better identify point sources with lower emissions under the same conditions. However, achieving the aim of 0.184 Mt CO_2_/yr is still very difficult to realize for space-based instruments with medium spatial resolution. For instance, even for a pixel size of 0.5 × 0.5 km^2^, the *ε_s_* needs be lower than 0.11 ppm if the emitted CO_2_ plume or XCO_2_ enhancement pixel can be detected by satellite. A notable improvement in detection capabilities is observed when the designed spatial resolution of satellite is enhanced from 2 km to 1 km. Specifically, the ΔXCO2_max_ values increase by ~0.5, ~0.7, ~1.1 and ~2.2 ppm for emissions of 3.6, 5.1, 7.3 and 14.5 Mt CO_2_/yr, respectively. For a satellite with an *ε_s_* of ~1 ppm and spatial resolutions of 2 km, 1 km, or 0.5 km, it could potentially detect power plants with emissions of 7.3, 3.6 or 1.56 Mt CO_2_/yr, respectively. But for a satellite with a spatial resolution of 4 km, it can only detect large-sized power plants with CO_2_ emissions exceeding 14.5 Mt CO_2_/yr.

### 3.3. Detection Capability of Satellite under Different Wind Field Conditions

**Figure 4:**
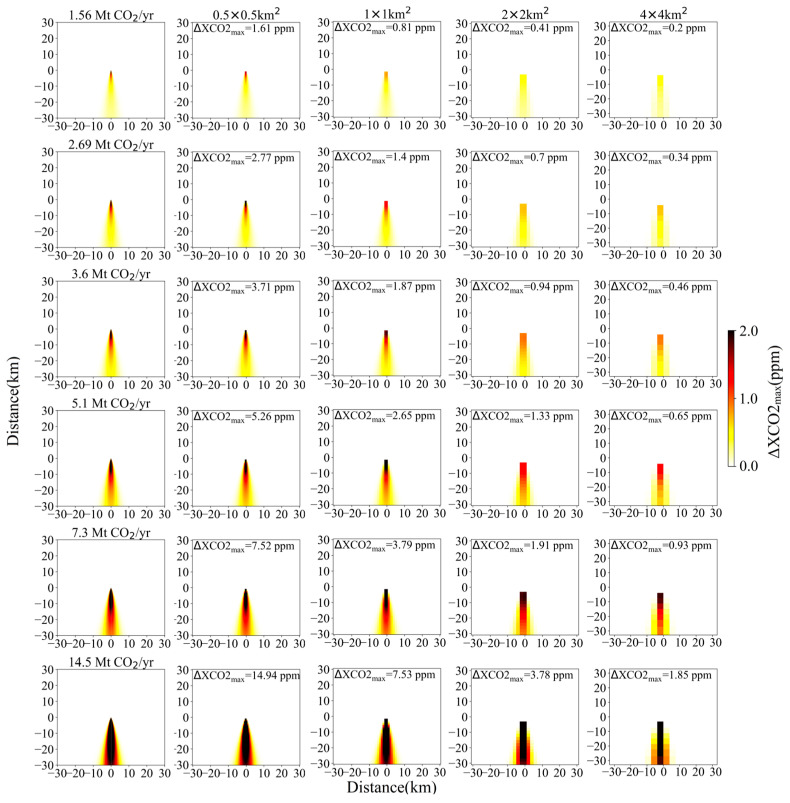
XCO2 enhancement distribution and ΔXCO2_max_ simulations with μ = 2 m/s and θ = 270°. Column 1 shows the simulated Gaussian plume at 50 m resolution for each power plant emission rate against a clean background. Columns 2~5 are simulated observations with a spatial resolution of 0.5, 1, 2 and 4 km, respectively. Top to bottom rows are simulated observations for CO_2_ emissions of 0.184, 1.56, 2.69, 3.6, 5.1, 7.3 and 14.5 Mt CO_2_/yr, respectively.

**Figure 5:**
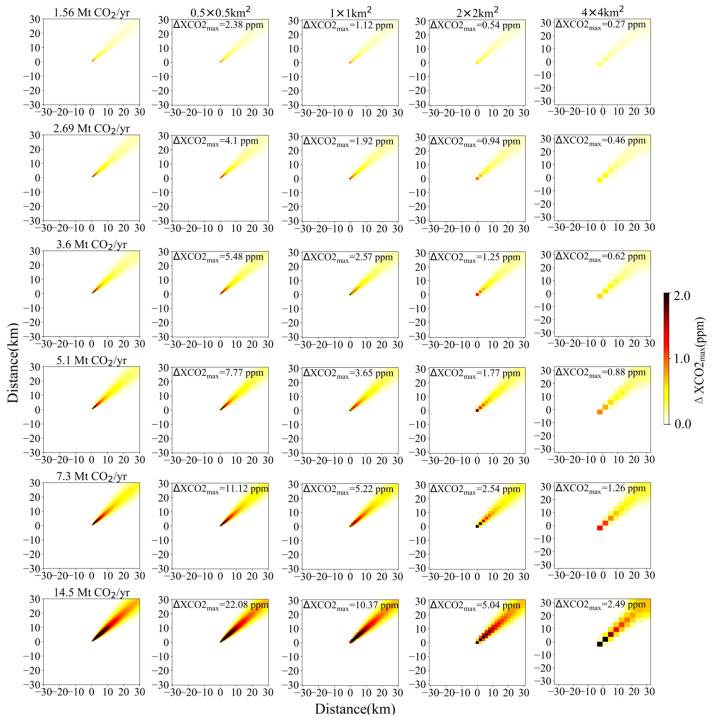
XCO_2_ enhancement distribution and ΔXCO2_max_ simulations with *μ* = 4 m/s and *θ* = 45°. Column 1 shows the simulated Gaussian plume at 50 m resolution for each power plant emission rate against a clean background. Columns 2~5 are simulated observations with a spatial resolution of 0.5, 1, 2 and 4 km, respectively. Top to bottom rows are simulated observations for CO_2_ emissions of 0.184, 1.56, 2.69, 3.6, 5.1, 7.3 and 14.5 Mt CO_2_/yr, respectively.

Compared with power plant emission and satellite-derived XCO_2_ precision which are considered relatively stable factors, the wind field exhibits continuous temporal and spatial variability. During the diffusion process of CO_2_ plume, the wind speed not only affects the propagation speed of the plume but also has an impact on the XCO_2_ gradient within the plume. Consequently, for a relatively fixed CO_2_ emission rate of the power plant, whether the emitted CO_2_ plume can be identified by satellite mostly depends on both the instantaneous wind speed and wind direction. As shown in Figure 4 and Figure 5 which are similar to Figure 3 with different wind speed and wind direction, the ΔXCO2_max_ varies from 0.23 to 13.18 ppm for a certain parameter set of CO_2_ emission level and satellite spatial resolution. With an increased emission amount and satellite spatial resolution, the impact of wind field on the variation of ΔXCO2_max_ tends to become stronger. As for a spatial resolution of 2 km and a fixed *θ* of 270°, the ΔXCO2_max_ increases by 0.19 to 1.78 ppm as the *μ* decreases from 4 m/s to 2 m/s. Similarly, altering the *θ* from 270° to 45°, results in the ΔXCO2_max_ increase by 0.32~3.02 ppm for a fixed *μ* of 4 m/s. For a satellite with an *ε_s_* of 0.7 ppm and a spatial resolution of 2 km, it can distinguish a power plant with emission of 5.1 Mt CO_2_/yr from the background noise (as shown in Figure 3) at a *μ* of 4 m/s, but its detection capability can be enhanced to 2.69 Mt CO_2_/yr when the *μ* decreases to 2 m/s.

**Figure 6:**
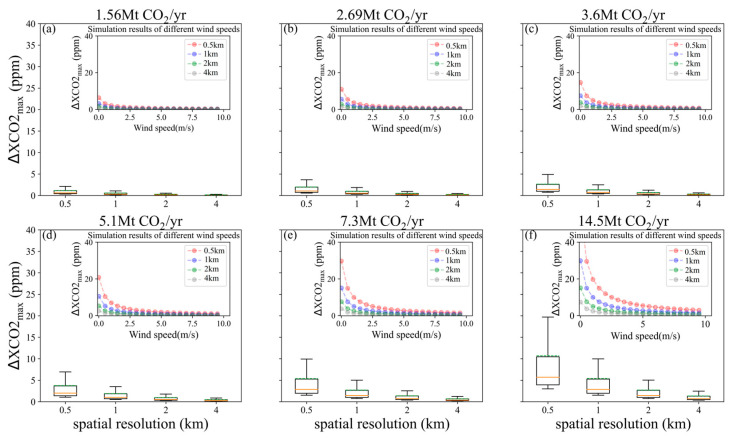
Box plots and line charts of the ΔXCO2_max_ simulation results for *θ* = 270° and *μ* from 0.5 to 10 m/s. (**a**–**f**) are corresponding to emissions of 1.56, 2.69, 3.6, 5.1, 7.3 and 14.5 Mt CO_2_/yr, the green dotted and orange lines represent the mean and median, respectively.

**Figure 7:**
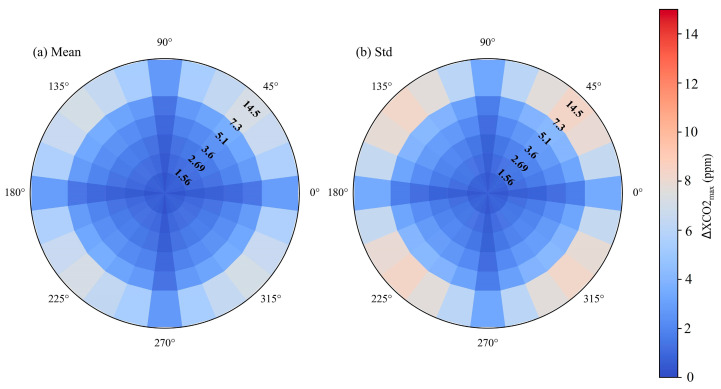
Means (**a**) and standard deviations (**b**) of ΔXCO2_max_ for fixed *θ* and different *μ* values (0.5~10 m/s) in polar coordinates.

In order to explore the relationship between the wind field and the satellite capability for the CO_2_ plume detection, XCO_2_ distribution and ΔXCO2_max_ were calculated by varying the *μ* from 0.5 m/s to 10 m/s in increments of 0.5 m/s and the *θ* from 0° to 360° in increments of 15°. Based on the statistics data of ΔXCO2_max_ in Figure 6, ΔXCO2_max_ is inversely proportional to both satellite spatial resolution and *μ.* ΔXCO2_max_ rapidly reduces with *μ* increasing for *μ* within the range of 1.5 and 5 m/s, while this downward trend slows down for *μ* greater than 5 m/s. Figure 7 presents the mean and standard deviation results of ΔXCO2_max_ from XCO_2_ distribution simulations, displayed in polar coordinates for a fixed *θ* across varying *μ* values. And the concentric rings, from inner to outer, correspond to the ΔXCO2_max_ simulations for emission levels of 1.56, 2.69, 3.6, 5.1, 7.3 and 14.5 Mt CO_2_/yr, respectively. The results indicate that for the wind directions at 45°, 135°, 225° and 315°, both the means and standard deviations of ΔXCO2_max_ are significantly larger than those for the wind directions at 0°, 90°, 180° and 270°. The main reason is that a greater number of valid 50 m × 50 m grids containing high XCO_2_ data within the CO_2_ plume image is involved in calculating averaged XCO_2_ enhancements when *θ* is set to 45°, 135°, 225° or 315°.

**Figure 8:**
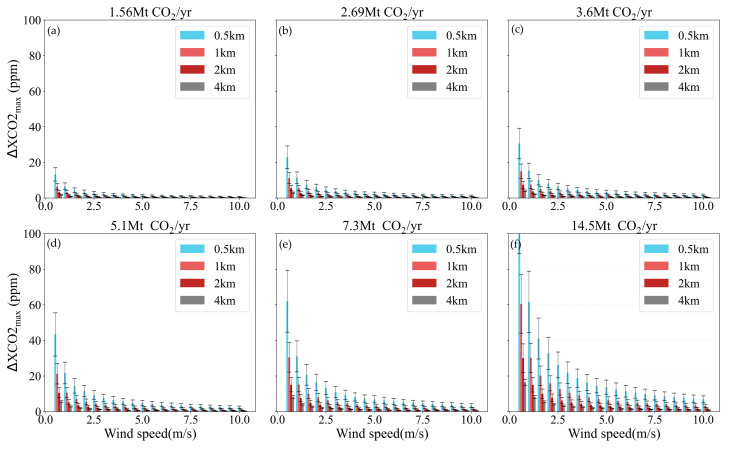
ΔXCO2_max_ means with fixed *μ* and different *θ* values (0°~360°) for different spatial resolutions of satellite. Error bars show the standard deviations of ΔXCO2_max_ in the binned *μ.* (**a**–**f**) are corresponding to emissions of 1.56, 2.69, 3.6, 5.1, 7.3 and 14.5 Mt CO_2_/yr, respectively.

Figure 8 describes the variation in ΔXCO2_max_ with the increase in *μ* for different power plant emission levels and satellite spatial resolutions. The error bars are corresponding to the standard deviations of ΔXCO2_max_ for all θ values ranging from 0° to 360° in 5°increments, within each binned *μ* category. For small-sized power plants (emissions < 6 Mt CO_2_/yr) and a pixel size of 2 × 2 km^2^, the variation in ΔXCO2_max_ among different wind directions is very small (<1 ppm) when the *μ* is larger than 3 m/s, and such difference declines as the wind speed increases. However, for the *μ* below 1.5 m/s, the difference in ΔXCO2_max_ among different wind directions can reach up to about 4 to 8 ppm. The ΔXCO2_max_ standard deviation increases for the power plants with higher emission levels (e.g., mid-sized power plants), meaning a strong impact of wind direction on the ΔXCO2_max_. Due to the positive correlation between the pixel size and the satellite-detected ability of CO_2_ plume, this trend is further accentuated for smaller pixel sizes, such as 0.5 km and 1 km, where a remarkable growth appears in the ΔXCO2_max_ standard deviation is observed. Therefore, both wind speed and wind direction are critical parameters which can introduce large uncertainty into CO_2_ emission estimation by using satellite-derived XCO_2_, especially for power plants at medium/high emission levels.

## 4. Uncertainty Analysis

### 4.1. Impact of the Uncertainty in Wind Field

Here, a 10% uncertainty was assumed for both wind speed and wind direction which can be obtained from atmospheric reanalysis or numerical weather prediction data [34]. The uncertainty of the wind field *ε_w_* is quantified as the standard deviation of the ΔXCO2_max_ variation, which was calculated by separately adding a random error of 10% in both wind speed and wind direction for each plume simulation. The parameters used for these CO_2_ plume simulations were consistent with those in Section 3.3, ensuring uniformity and comparability across the study.

**Figure 9:**
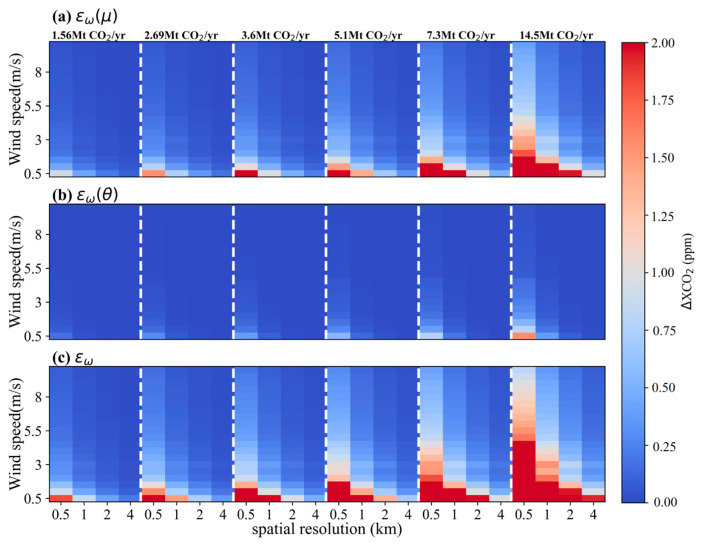
The simulated uncertainties of ΔXCO_2_ associated with the wind field for *θ =* 45°. (**a**), (**b**) and (**c**) are for *ε_w_*(*μ*), *ε_w_*(*θ*) and *ε_w_*, respectively.

**Figure 10:**
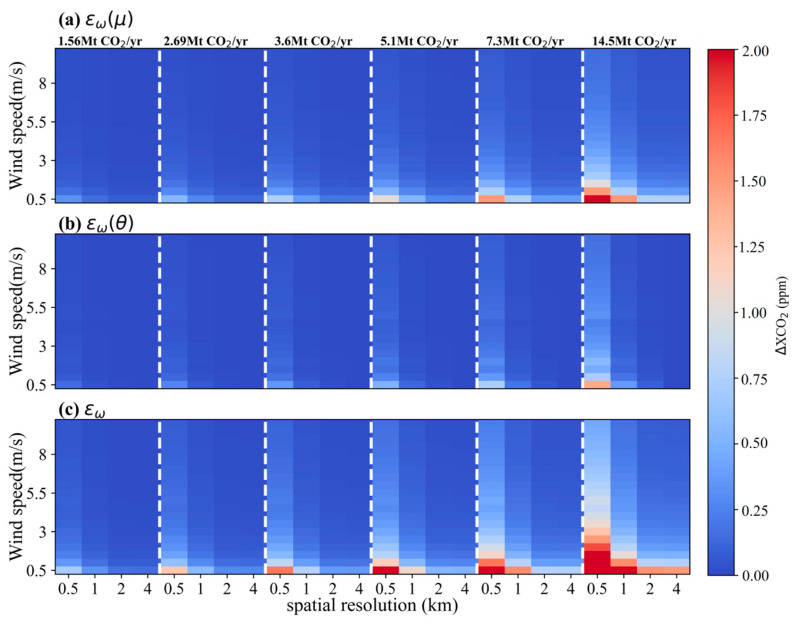
The simulated uncertainties of ΔXCO_2_ associated with the wind field for *θ =* 270°. (**a**), (**b**) and (**c**) are for *ε_w_*(*μ*), *ε_w_*(*θ*) and *ε_w_*, respectively.

The uncertainties of satellite-detected XCO_2_ enhancement led by errors of wind field under scenarios of *θ* = 45° and 270° are shown in Figure 9 and Figure 10, respectively. Generally, compared to the impact of *ε_w_*(*θ*), *ε_w_*(*μ*) plays a dominant role in the variation of *ε_w_* for both *θ* = 45° and 270°. And the uncertainties of ΔXCO_2_ caused by the uncertainty in wind speed is more significant than those introduced from the uncertainty in wind direction. Under a condition with the same wind speed and emission level, there is a significant difference in *ε_w_*(*μ*) and *ε_w_* between *θ* = 45° and *θ* = 270° scenarios, while the *ε_w_*(*θ*) at *θ* = 45° is similar to that at *θ* = 270°. For example, in the case of a small-sized power plant with a CO_2_ emission of 5.1 Mt CO_2_/yr, *ε_w_*(*μ*) values vary in the range of 0.36~2.80 ppm and 0.17~1.37 ppm for satellite pixel sizes of 1 × 1 km^2^ and 2 × 2 km^2^, respectively, with *θ* = 45° and *μ* ranging from 0.5 to 4 m/s. Under the same conditions yet at *θ* = 270°, *ε_w_*(*μ*) only spans from 0.13 to 1.06 ppm and 0.06 to 0.53 ppm for the satellite with spatial resolutions of 1 km and 2 km, respectively. In comparison with *ε_w_*(*μ*), *ε_w_*(*θ*) is smaller and its variation is more stable. And the *ε_w_*(*θ*) values at both *θ* = 45° and 270° are all less than 0.5 ppm. As for the overall uncertainty of ΔXCO_2_ arising from both wind speed and wind direction, the maximum difference in *ε_w_* between *θ* = 45° and *θ* = 270° is approximately 2.5 times under conditions of point sources emitting less than 6 Mt CO_2_/yr, *μ* below 2 m/s and spatial resolution coarser than 1 km. Taking the power plant with CO_2_ emission of 5.1 Mt CO_2_/yr at *μ* = 0.5 m/s as an example, *ε_w_* values at *θ* = 45° reach up to 2.81 and 1.37 ppm corresponding to satellite spatial resolution of 1 km and 2 km, respectively, whereas they are only 1.09 and 0.54 ppm for *θ* = 270°. When the *μ* increases to 4 m/s under these conditions, there is a significant decline in *ε_w_*. These results at *θ* = 45° are 0.36 and 0.17 ppm for 1 km and 2 km spatial resolutions, respectively, and are 0.14 and 0.06 ppm at *θ* = 270°. Such difference grows with increasing emission rates from the point source and decreases with rising wind speeds.

**Figure 11:**
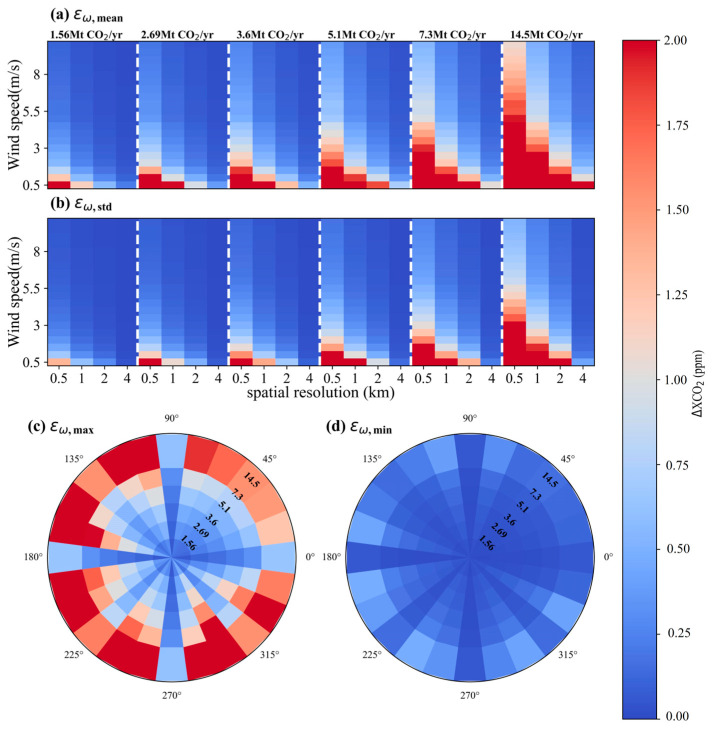
Statistical results of the simulated uncertainties in ΔXCO_2_ associated with the wind field. (**a**) and (**b**) are for means and standard deviations of *ε_w_* values at different wind speeds, respectively. (**c**) and (**d**) are for maximums and minimums of *ε_w_* at different wind directions, respectively.

To comprehensively investigate the influence of uncertainties in wind speed and wind direction on satellite detection of CO_2_ plumes, simulations similar to those in Figure 9 and Figure 10 were extended to *θ* ranging from 0° to 360° with intervals of 15°. Figure 11a and Figure 11b are the means (*ε_w,mean_*) and standard deviations (*ε_w,std_*) of *ε_w_* values across all wind directions, respectively, for different wind speeds, pixel sizes and emission levels. Both *ε_w,mean_* and *ε_w,std_* are directly proportional to the CO_2_ emission rate of the power plant, while inversely correlating with the spatial resolution of satellite. For a point source emitting 5.1 Mt CO_2_/yr, the *ε_w_* decreases from 3.75 ± 2.01 ppm (expressed as *ε_w,mean_* ± *ε_w,std_*) to 0.46 ± 0.24 ppm with *μ* increasing from 0.5 m/s to 4 m/s for a pixel size of 1 × 1 km^2^. Similarly, *ε_w_* reduces from 1.82 ± 0.95 ppm to 0.22 ± 0.11 ppm when the pixel size is downscaled to 2 × 2 km^2^. Figure 11c and 11d illustrate the maximums (*ε_w,max_*) and minimums (*ε_w,min_*) of *ε_w_* across all wind speeds in the range of 0.5~10 m/s for a satellite spatial resolution of 2 km on different wind directions, respectively, where *ε_w,max_* = *ε_w,mean_* + *ε_w,std_* and *ε_w,min_* = *ε_w,mean_*-*ε_w,std_*. The results indicate a more significant difference in *ε_w,max_* compared to that of *ε_w,min_* among different wind directions. For a small-sized power plant with a CO_2_ emission of 5.1 Mt CO_2_/yr, the *ε_w,max_* across wind directions from 0° to 360° varies from 0.21 ppm to 1.56 ppm with an average of 0.71 ppm, and the *ε_w,min_* ranges only from 0.02 ppm to 0.15 ppm with an average of 0.08 ppm. As the emission rate of the power plant increases, the range of variability also increases. For a large-sized power plant with a CO_2_ emission of 14.5 Mt CO_2_/yr, the *ε_w,max_* spans from 0.61 ppm to 4.45 ppm, averaging 2.03 ppm, and the *ε_w,min_* ranges from 0.05 to 0.47 ppm, averaging 0.2 ppm.

### 4.2. Impact of the Uncertainty in CO_2_ Emission Level

**Figure 12:**
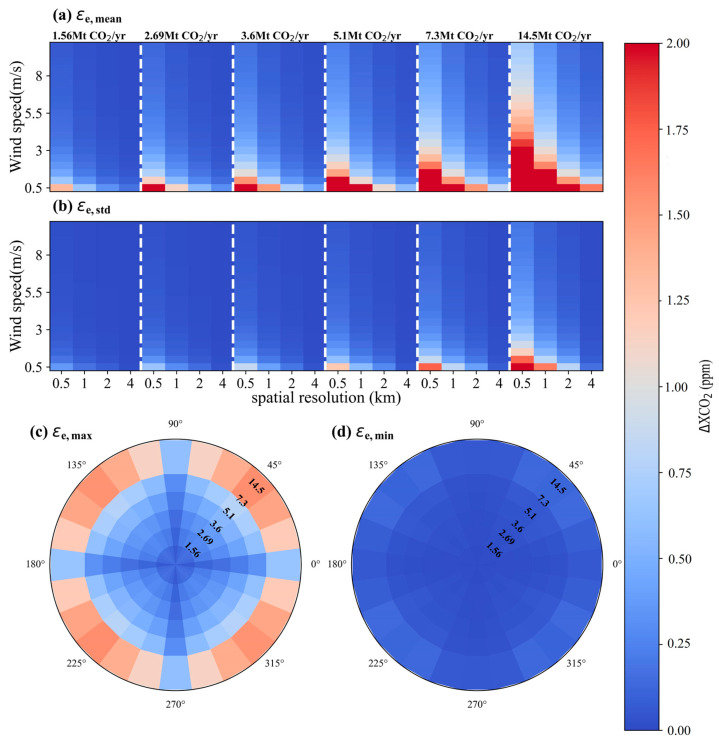
Statistical results of the simulated uncertainties in ΔXCO_2_ associated with the wind field. (**a**) and (**b**) are for means and standard deviations of *ε_e_* values at different wind speeds, respectively. (**c**) and (**d**) are for maximums and minimums of *ε_e_* at different wind directions, respectively.

Developed and developing countries are gradually adopting continuous emission monitoring systems (CEMS) to monitor the real-time carbon emissions from thermal power plants, in order to obtain accurate, complete, and timely emission data. CEMS data are also uncertain, but we assume that those uncertainties are small, i.e., <10% [35,36]. Similar to the analysis of *ε_w_*, an uncertainty of 10% was assumed for the CO_2_ emission from power plants. Therefore, *ε_e_* results were calculated by introducing a random error of 10% into the CO_2_ emission rate for each plume simulation. The *ε_e_* variations were further examined under different wind speeds and wind directions, as shown in Figure 12. Figure 12a and Figure 12b give the means (*ε_e__,mean_*) and standard deviations (*ε_e__,std_*) of *ε_e_* values at all wind directions, respectively, for various wind speeds, pixel sizes and emission levels. The variability in *ε_e_* among different wind directions is obvious only at low wind speeds. For instance, for a pixel size of 1 × 1 km^2^, the *ε_e_* varies from 0.11 ± 0.03 ppm to 6.04 ± 1.63 as the *μ* decreases from 3 m/s to 0.5 m/s, while it correspondingly ranges from 0.05 ± 0.01 ppm to 3.00 ± 0.8 ppm for 2 × 2 km^2^. The maximums (*ε_e__,max_*) and minimums (*ε_e__,min_*) of *ε_e_* under all scenarios with *μ* between 0.5 to 10 m/s are shown in Figure 12c and 12d, respectively, for a satellite spatial resolution of 2 km. The results reveal that the *ε_e_* variability due to different wind speeds is closely related to wind direction and exhibits strong regularity. Both *ε_e__,max_* and *ε_e__,min_* values are relatively small, with *ε_e__,max_* and *ε_e__,mmin_* results of different CO_2_ emission levels below 1.55 ppm and 0.5 ppm, respectively.

### 4.3. Estimation of Overall Uncertainty

**Figure 13:**
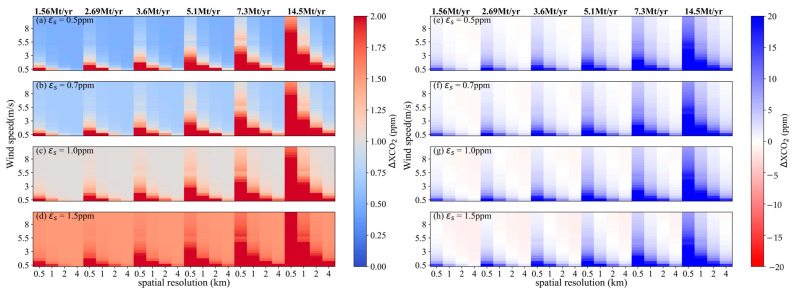
Overall uncertainties in satellite-detected XCO_2_ enhancement and the difference between them and ΔXCO2_max_ for *θ* = 45°. (**a**–**d**) are *ε* values calculated from *ε_s_* = 0.5, 0.7, 1.0 and 1.5 ppm, respectively. (**e**–**h**) are results of ΔXCO2_max_ subtracted from *ε* for *ε_s_* = 0.5, 0.7, 1.0 and 1.5 ppm, respectively.

**Figure 14:**
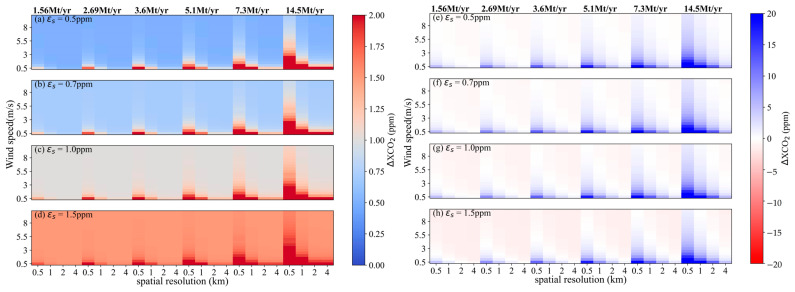
Overall uncertainties in satellite-detected XCO_2_ enhancement and the difference between them and ΔXCO2_max_ for *θ* = 270°. (**a**–**d**) are *ε* values calculated from *ε_s_* = 0.5, 0.7, 1.0 and 1.5 ppm, respectively. (**e**–**h**) are results of ΔXCO2_max_ subtracted from *ε* for *ε_s_* = 0.5, 0.7, 1.0 and 1.5 ppm, respectively.

The overall uncertainties in satellite-detected XCO_2_ enhancement were derived using Equation (4). Here, the satellite-based XCO_2_ error, *ε_s_*, were set at varying levels of 0.5, 0.7, 1.0 and 1.5 ppm. The calculations of *ε_w_* and *ε_e_* correspond to the results in Section 4.1 and Section 4.2, respectively. The overall uncertainties *ε* with different *ε_s_* values under conditions of *θ* = 45° are shown in Figure 13a–d, and results for *θ* = 270° are correspondingly depicted in Figure 14a–d. The variation trend of *ε* influenced by changes in wind speed, CO_2_ emission level and satellite pixel size are similar for both *θ* = 45° and *θ* = 270°, but *ε* calculated for *θ* = 45° is larger than that for *θ* = 270° under the same scenario. The wind field significantly influences the variation of *ε*. Specifically, for an *ε_s_* = 0.7 ppm and a power plant with an emission of 5.1 Mt CO_2_/yr, *ε* simulations on 1 × 1 km^2^ grids vary in the range of 0.74~4.0 ppm with an average of 1.09 ppm when *θ* = 45° and *μ* ranging from 0.5 to 4 m/s. And *ε* results range in the range of 0.70~2.0 ppm with an average of 0.83 ppm for 2 × 2 km^2^ pixel size. On the wind direction of 270°, the *ε* range for a 1 × 1 km² pixel size is 0.7 to 1.67 ppm, averaging 0.79 ppm, and for a spatial resolution of 2 km, it is 0.70 to 1.03 ppm, averaging 0.72 ppm. When the wind speed is larger than 4 m/s, *ε* is almost constant for a pixel size bigger than 2 × 2 km^2^.

To further investigate the performance of satellite in detecting CO_2_ plumes from power plants with different CO_2_ emissions, results of ΔXCO2_max_ subtracted from *ε* were analyzed, as shown in Figure 13e–h and Figure 14e–h. Here, blue regions represent the overall uncertainty less than ΔXCO2_max_, meaning that satellite can detect the signal of XCO_2_ enhancements generated by power plant emissions. White and light red regions suggest a reduced likelihood of satellite detection for CO_2_ emission plumes. The detection capability of satellite under *θ* = 45° is proved more effective than that under *θ* = 270°. Generally, it is easier for satellite to detect XCO_2_ enhancements when wind speed is below 3 m/s, particularly for medium- and large-sized power plants. In order to capture the CO_2_ emission from small-sized power plants, the spatial resolution of the satellite should be better designed to be finer than 1 km. The probability of detecting CO_2_ plume significantly decreases as the pixel size increases, and it depends to a large extent on the meteorologic condition.

## 5. Summary

The spatial resolution and the precision of satellite-derived XCO_2_ measurements are two key indicators for the payload design of the next-generation CO_2_ satellite missions, which decide the satellite’s capability in detecting CO_2_ emissions. In this paper, by using the high-resolution Gaussian model, we focused on the simulation and uncertainty factors in satellite detection of CO_2_ emissions from power plants on different conditions of satellite pixel sizes, satellite-derived XCO_2_ errors and CO_2_ emission levels. According to our simulations, detailed conclusions on the detection capabilities of satellites and their associated uncertainties in monitoring CO_2_ plumes are found:

(1) Generally, enhanced spatial resolution of satellite, coupled with improved precision in space-based XCO_2_ measurements will be crucial in accurately detecting and quantifying CO_2_ emissions from various-sized power plants under diverse meteorological conditions. For low-level emissions (6 Mt CO_2_/yr), detecting CO_2_ plumes becomes challenging when the satellite spatial resolution is coarser than 1 km, because of its CO_2_ enhancement being comparable to satellite observational errors. A reduction in the satellite-retrieved XCO_2_ precision from 1.5 ppm to 0.7 ppm can significantly enhance the detection capabilities. For high emission levels (25 Mt CO_2_/yr), the plumes are just observable at pixel sizes of 0.5, 1, and 2 km for *ε_s_* = 1.5 ppm. However, for *ε_s_* = 0.7 ppm, power plants with emissions greater than 13 Mt CO_2_/yr could be detected with a spatial resolution of 4 km or higher.
(2) Wind speed and wind direction significantly impact the detectability of CO_2_ plumes for satellites. Variations in wind conditions lead to substantial differences in the maximum detectable CO_2_ enhancement. For a satellite by an *ε_s_* of 0.7 ppm and a spatial resolution of 2 km, it is capable of differentiating a power plant with an emission of 5.1 Mt CO_2_/yr from ambient background noise at a wind speed of 4 m/s. Notably, this detection threshold is further improved when the wind speed is reduced to 2 m/s, allowing for the identification of power plants with an emission of 2.69 Mt CO_2_/yr.
(3) Our results indicate that *ε_w_*(*μ*) plays a dominant role in the variation of simulated XCO_2_ uncertainty introduced by uncertainties in both wind speed and wind direction. With the assumption of a 10% uncertainty in both wind speed and wind direction, for a small-sized power plant (5.1 Mt CO_2_/yr) under conditions of wind speed increasing from 0.5 m/s to 4 m/s, the *ε_w_* reduces from 3.75 ± 2.01 ppm to 0.46 ± 0.24 ppm for 1 × 1 km^2^ pixel size, while it changes from 1.82 ± 0.95 ppm to 0.22 ± 0.11 ppm for 1 × 1 km^2^. These variations highlight the sensitivity of satellite detection capabilities to meteorological conditions.
(4) The overall uncertainty in satellite-detected XCO_2_ enhancement was calculated using a combination of uncertainties in wind field, satellite-derived XCO_2_ error, and CO_2_ emission levels, considering various *ε_s_* levels (0.5, 0.7, 1.0, and 1.5 ppm) and 10% uncertainty for obtained wind field and CO_2_ emission data. The results indicate that *ε* is significantly influenced by the wind field. Although *ε* for *θ* = 45° is larger than that for *θ* = 270° under the same scenario, satellite has a more effective capability for detecting CO_2_ emission at *θ* = 45° due to a more rapid growth of ΔXCO2_max_. A designed spatial resolution of satellite better than 1 km is suggested, because the CO_2_ emission from small-sized power plants is much more likely be detected when the wind speed is below 3 m/s.

## 6. Conclusions

In designing satellite payloads, our approach involves forward simulation of plume dispersion at specific spatial resolutions, allowing us to assess whether these results meet the monitoring precision requirements set by the satellite. Once the satellite’s resolution and monitoring precision are confirmed, further refinement of parameters such as signal-to-noise ratio, sampling rate, and spectral resolution will continue. Throughout the simulation process, we strive to incorporate meteorological factors that influence plume dispersion in real-world scenarios. By leveraging scientifically simulated outcomes, this study offers practical insights for refining payload specifications to meet the demands of scientific missions and achieve effective pollution monitoring results.

This research holds significant implications for the design and operation of future CO_2_ monitoring satellites, such as the Chinese Tansat2. By focusing on simulating plume dispersion based on satellite spatial resolution and monitoring precision, we explore the satellite’s capability to identify plumes under various conditions. With a monitoring precision of 0.7 ppm, the satellite demonstrates effective capturing of emissions from small to medium-scale ground point sources. Through simulations of point sources with different emission capacities and consideration of wind errors, point source emission rate errors, and satellite monitoring errors, we conduct a quantitative analysis of plume simulation accuracy at different resolutions. Our findings indicate that under typical wind speed conditions, a satellite spatial resolution of 1 km enables effective monitoring of ground point source emissions.

The quantitative analysis of uncertainties and detection capabilities across different scenarios serves as a foundation for future advancements in satellite monitoring of anthropogenic CO_2_ emissions, essential for addressing challenges related to climate change. Subsequent work could focus on refining these simulations with actual satellite data and extending the analysis using complex models to simulate more realistic CO_2_ plumes.

## Data Availability

The data presented in this study are available on request from the corresponding author. The data are not publicly available due to restrictions and privacy.
